# Supplementary material for: HIVprotI: an integrated web based platform for prediction and design of HIV proteins inhibitors
Source: J Cheminform. 2018 Mar 9;10:12. doi: 10.1186/s13321-018-0266-y (PMC5845081; doi:10.1186/s13321-018-0266-y)
Supplement: Supplementary file 2 — Additional file 2. Source code of HIVProtI web server. [file 13321_2018_266_MOESM2_ESM.zip › HIVprotI_Source-code/hivprotI/weka-3-6-6/changelogs/CHANGELOG-3-5-2.html]

Weka-3-5-2 Changelog


# Weka-3-5-2 Changelog

Designed for use with Ant.

---

2005-12-22 01:02 eibe

```
Added a few more options.
```

- filters/unsupervised/attribute/StringToWordVector.html (1.10)

2005-12-19 05:21 fracpete

```
fixed Javadoc for gr-method
```

- core/Utils.html (1.49)

2005-12-17 23:17 fracpete

```
references to GenericObjectEditor removed since only GUI components need the GOE and they must perform a "GenericObjectEditor.registerEditors();" anyway
```

- classifiers/bayes/BayesNet.html (1.25)

2005-12-12 21:58 mhall

```
Minimum expected cost option has been broken since 2001clear
```

- classifiers/CostMatrix.html (1.10)

2005-12-11 19:48 fracpete

```
added support for average per column
```

- gui/experiment/Experimenter.props (1.3)
- gui/experiment/ExperimenterDefaults.html (1.3)
- gui/experiment/OutputFormatDialog.html (1.5)
- gui/experiment/ResultsPanel.html (1.39)

2005-12-11 19:46 fracpete

```
added support for average per column
```

- experiment/ResultMatrix.html (1.6)
- experiment/ResultMatrixLatex.html (1.4)
- experiment/ResultMatrixPlainText.html (1.6)
- experiment/ResultMatrixSignificance.html (1.3)

2005-12-07 03:50 mhall

```
Now includes an option for conservative selection in forward mode (i.e. will continue to add attributes as long as the merit does not decrease).
```

- attributeSelection/GreedyStepwise.html (1.6)

2005-12-06 22:05 cvs\_rrb

```
Allow reading in from XMLBIFF file where the nodes are not in sequential order.
```

- classifiers/bayes/net/BayesNetGenerator.html (1.8)

2005-12-05 22:00 fracpete

```
fix from Julien Nioche (J.Nioche at sheffield.ac.uk) to speed up generation of large nominal attributes (10-15 times faster)
```

- core/Attribute.html (1.36)

2005-12-04 21:05 mhall

```
Removed some old code that is not used
```

- attributeSelection/AttributeSelection.html (1.39)

2005-12-03 23:14 fracpete

```
no longer craps out if it encounters a missing class in the GenericPropertiesCreator.props file (e.g. happened if Developer-props-file was run with the book-version of Weka), just outputs an error message on the console
```

- gui/GenericPropertiesCreator.html (1.8)

2005-12-02 03:10 fracpete

```
cleaned up imports
added save-option to right-click-menu in history to save reduced/transformed data
```

- gui/explorer/AttributeSelectionPanel.html (1.36)

2005-12-01 21:16 fracpete

```
filter for adding a unique ID to instances via a separate attribute
```

- filters/unsupervised/attribute/AddID.html (1.1)

2005-12-01 00:57 fracpete

```
superseded by SortedTableModel
```

- gui/TableMap.html (1.2)
- gui/TableSorter.html (1.2)

2005-12-01 00:56 fracpete

```
ArffSortedTableModel replaces ArffTableSorter
```

- gui/arffviewer/ArffPanel.html (1.3)
- gui/arffviewer/ArffTable.html (1.4)
- gui/arffviewer/ArffTableCellRenderer.html (1.2)
- gui/arffviewer/ArffTableModel.html (1.4)
- gui/arffviewer/ArffViewerMainPanel.html (1.2)

2005-12-01 00:56 fracpete

```
superseded by ArffSortedTableModel
```

- gui/arffviewer/ArffTableSorter.html (1.4)

2005-12-01 00:54 fracpete

```
supersedes ArffTableSorter
```

- gui/arffviewer/ArffSortedTableModel.html (1.1)

2005-12-01 00:53 fracpete

```
supersedes weka.gui.TableMap and weka.gui.TableSorter
```

- gui/SortedTableModel.html (1.1)

2005-12-01 00:51 fracpete

```
new icons
```

- gui/images/back.gif (1.1)
- gui/images/home.gif (1.1)
- gui/images/resize.gif (1.1)

2005-11-29 04:24 fracpete

```
superseded by weka.core.ClassDiscovery
```

- core/RTSI.html (1.6)

2005-11-29 04:23 fracpete

```
replaced weka.core.RTSI with weka.core.ClassDiscovery
```

- core/stemmers/SnowballStemmer.html (1.4)

2005-11-29 04:22 fracpete

```
replaced weka.core.RTSI with weka.core.ClassDiscovery
```

- gui/visualize/PrintableComponent.html (1.5)

2005-11-29 04:22 fracpete

```
replaced weka.core.RTSI with weka.core.ClassDiscovery
```

- gui/experiment/DatasetListPanel.html (1.17)

2005-11-29 04:21 fracpete

```
replaced weka.core.RTSI with weka.core.ClassDiscovery
```

- gui/GenericObjectEditor.html (1.46)

2005-11-29 04:20 fracpete

```
replacement for weka.core.RTSI
```

- core/ClassDiscovery.html (1.1)

2005-11-28 03:00 fracpete

```
fixed main method - instantiated an Agrawal data generator instead of LED24
```

- datagenerators/classifiers/classification/LED24.html (1.2)

2005-11-18 00:42 mhall

```
Reversed the meaning of the -L option. Now locally predictive attributes are included by default.
```

- attributeSelection/CfsSubsetEval.html (1.21)

2005-11-18 00:29 mhall

```
Changed so that if all the instances in the training data have equal weight then no resampling is done
```

- classifiers/meta/AttributeSelectedClassifier.html (1.20)

2005-11-14 03:07 eibe

```
Fixed bug in ResultMatrixPlainText, which meant that displayed counts could be out of line with corresponding dataset and results. Changed TTesters to not throw an exception when missing values are encountered.
```

- experiment/PairedCorrectedTTester.html (1.8)
- experiment/PairedTTester.html (1.32)

2005-11-01 00:20 mhall

```
Updates from Jeffrey
```

- gui/beans/icons/ArffLoader.gif (1.2)
- gui/beans/icons/ArffLoader\_animated.gif (1.2)
- gui/beans/icons/C45Loader.gif (1.2)
- gui/beans/icons/C45Loader\_animated.gif (1.2)
- gui/beans/icons/CSVLoader.gif (1.2)
- gui/beans/icons/CSVLoader\_animated.gif (1.2)
- gui/beans/icons/DefaultDataSink.gif (1.2)
- gui/beans/icons/DefaultDataSink\_animated.gif (1.2)
- gui/beans/icons/DefaultDataSource.gif (1.3)
- gui/beans/icons/DefaultDataSource\_animated.gif (1.3)
- gui/beans/icons/SerializedInstancesLoader.gif (1.2)
- gui/beans/icons/SerializedInstancesLoader\_animated.gif (1.2)

2005-11-01 00:19 mhall

```
branches:  1.1.2;
More new icons from Jeffrey
```

- gui/beans/icons/ArffSaver.gif (1.1)
- gui/beans/icons/ArffSaver\_animated.gif (1.1)
- gui/beans/icons/C45Saver.gif (1.1)
- gui/beans/icons/C45Saver\_animated.gif (1.1)
- gui/beans/icons/CSVSaver.gif (1.1)
- gui/beans/icons/CSVSaver\_animated.gif (1.1)
- gui/beans/icons/DatabaseSaver.gif (1.1)
- gui/beans/icons/DatabaseSaver\_animated.gif (1.1)
- gui/beans/icons/SerializedInstancesSaver.gif (1.1)
- gui/beans/icons/SerializedInstancesSaver\_animated.gif (1.1)

2005-11-01 00:13 mhall

```
More new icons from Jeffrey
```

- gui/beans/icons/DatabaseLoader.gif (1.1)
- gui/beans/icons/DatabaseLoader\_animated.gif (1.1)

2005-10-31 22:18 mhall

```
Yet more new and groovy icons from Jeffrey
```

- gui/beans/icons/ClassBalancedND.gif (1.1)
- gui/beans/icons/ClassBalancedND\_animated.gif (1.1)
- gui/beans/icons/DBScan.gif (1.1)
- gui/beans/icons/DBScan\_animated.gif (1.1)
- gui/beans/icons/Dagging.gif (1.1)
- gui/beans/icons/Dagging\_animated.gif (1.1)
- gui/beans/icons/DataNearBalancedND.gif (1.1)
- gui/beans/icons/DataNearBalancedND\_animated.gif (1.1)
- gui/beans/icons/END.gif (1.1)
- gui/beans/icons/END\_animated.gif (1.1)
- gui/beans/icons/GaussianProcesses.gif (1.1)
- gui/beans/icons/GaussianProcesses\_animated.gif (1.1)
- gui/beans/icons/HNB.gif (1.1)
- gui/beans/icons/HNB\_animated.gif (1.1)
- gui/beans/icons/LibSVM.gif (1.1)
- gui/beans/icons/LibSVM\_animated.gif (1.1)
- gui/beans/icons/OPTICS.gif (1.1)
- gui/beans/icons/OPTICS\_animated.gif (1.1)
- gui/beans/icons/XMeans.gif (1.1)
- gui/beans/icons/XMeans\_animated.gif (1.1)

2005-10-31 03:33 mhall

```
Small modification to allow loading of a default icon for a subpackage of classifiers
```

- gui/beans/Classifier.html (1.21)

2005-10-31 03:24 mhall

```
branches:  1.1.2;
More new icons from Jeffrey
```

- gui/beans/icons/Default\_bayesClassifier.gif (1.1)
- gui/beans/icons/Default\_bayesClassifier\_animated.gif (1.1)
- gui/beans/icons/Default\_functionsClassifier.gif (1.1)
- gui/beans/icons/Default\_functionsClassifier\_animated.gif (1.1)
- gui/beans/icons/Default\_lazyClassifier.gif (1.1)
- gui/beans/icons/Default\_lazyClassifier\_animated.gif (1.1)
- gui/beans/icons/Default\_metaClassifier.gif (1.1)
- gui/beans/icons/Default\_metaClassifier\_animated.gif (1.1)
- gui/beans/icons/Default\_miscClassifier.gif (1.1)
- gui/beans/icons/Default\_miscClassifier\_animated.gif (1.1)

2005-10-31 03:24 mhall

```
More new icons from Jeffrey
```

- gui/beans/icons/Default\_nestedDichotomiesClassifier.gif (1.1)

2005-10-31 03:24 mhall

```
More new icons from Jeffrey
```

- gui/beans/icons/Default\_nestedDichotomiesClassifier\_animated.gif (1.1)

2005-10-31 03:24 mhall

```
branches:  1.1.2;
More new icons from Jeffrey
```

- gui/beans/icons/Default\_rulesClassifier.gif (1.1)
- gui/beans/icons/Default\_rulesClassifier\_animated.gif (1.1)
- gui/beans/icons/Default\_treeClassifier.gif (1.1)
- gui/beans/icons/Default\_treeClassifier\_animated.gif (1.1)

2005-10-31 03:23 mhall

```
Update from Jeffrey
```

- gui/beans/icons/DefaultAssociator.gif (1.2)
- gui/beans/icons/DefaultAssociator\_animated.gif (1.2)

2005-10-30 06:01 mhall

```
Modifications to accomodate Carsten Pohle's web log processing components
```

- gui/beans/Filter.html (1.14)
- gui/beans/Loader.html (1.13)

2005-10-30 05:56 mhall

```
Initial import
```

- gui/beans/ConnectionNotificationConsumer.html (1.1)
- gui/beans/LogWriter.html (1.1)

2005-10-28 02:12 mhall

```
branches:  1.1.2;
Fixed naming bug
```

- gui/beans/icons/ConjunctiveRule\_animated.gif (1.1)

2005-10-28 02:10 mhall

```
Naming bug
```

- gui/beans/icons/ConjunctiveRule\_animted.gif (1.2)

2005-10-28 02:03 mhall

```
branches:  1.1.2;
More new icons from Jeffrey
```

- gui/beans/icons/ConjunctiveRule.gif (1.1)
- gui/beans/icons/DecisionTable.gif (1.1)
- gui/beans/icons/DecisionTable\_animated.gif (1.1)
- gui/beans/icons/JRip.gif (1.1)
- gui/beans/icons/JRip\_animated.gif (1.1)
- gui/beans/icons/M5Rules.gif (1.1)
- gui/beans/icons/M5Rules\_animated.gif (1.1)
- gui/beans/icons/NNge.gif (1.1)
- gui/beans/icons/NNge\_animated.gif (1.1)
- gui/beans/icons/OneR.gif (1.1)
- gui/beans/icons/OneR\_animated.gif (1.1)
- gui/beans/icons/PART.gif (1.1)
- gui/beans/icons/PART\_animated.gif (1.1)
- gui/beans/icons/Prism.gif (1.1)
- gui/beans/icons/Prism\_animated.gif (1.1)
- gui/beans/icons/ZeroR.gif (1.1)
- gui/beans/icons/ZeroR\_animated.gif (1.1)

2005-10-28 01:46 mhall

```
branches:  1.1.2;
New icons from Jeffrey
```

- gui/beans/icons/AdaBoostM1.gif (1.1)
- gui/beans/icons/AdaBoostM1\_animated.gif (1.1)
- gui/beans/icons/AdditiveRegression.gif (1.1)
- gui/beans/icons/AdditiveRegression\_animated.gif (1.1)
- gui/beans/icons/AttributeSelectedClassifier.gif (1.1)
- gui/beans/icons/AttributeSelectedClassifier\_animated.gif (1.1)
- gui/beans/icons/Bagging.gif (1.1)
- gui/beans/icons/Bagging\_animated.gif (1.1)
- gui/beans/icons/CVParameterSelection.gif (1.1)
- gui/beans/icons/CVParameterSelection\_animated.gif (1.1)
- gui/beans/icons/ClassificationViaRegression.gif (1.1)
- gui/beans/icons/ClassificationViaRegression\_animated.gif (1.1)
- gui/beans/icons/CostSensitiveClassifier.gif (1.1)
- gui/beans/icons/CostSensitiveClassifier\_animated.gif (1.1)
- gui/beans/icons/Decorate.gif (1.1)
- gui/beans/icons/Decorate\_animated.gif (1.1)
- gui/beans/icons/FilteredClassifier.gif (1.1)
- gui/beans/icons/FilteredClassifier\_animated.gif (1.1)
- gui/beans/icons/Grading.gif (1.1)
- gui/beans/icons/Grading\_animated.gif (1.1)
- gui/beans/icons/LogitBoost.gif (1.1)
- gui/beans/icons/LogitBoost\_animated.gif (1.1)
- gui/beans/icons/MetaCost.gif (1.1)
- gui/beans/icons/MetaCost\_animated.gif (1.1)
- gui/beans/icons/MultiBoostAB.gif (1.1)
- gui/beans/icons/MultiBoostAB\_animated.gif (1.1)
- gui/beans/icons/MultiClassClassifier.gif (1.1)
- gui/beans/icons/MultiClassClassifier\_animated.gif (1.1)
- gui/beans/icons/MultiScheme.gif (1.1)
- gui/beans/icons/MultiScheme\_animated.gif (1.1)
- gui/beans/icons/OrdinalClassClassifier.gif (1.1)
- gui/beans/icons/OrdinalClassClassifier\_animated.gif (1.1)
- gui/beans/icons/RacedIncrementalLogitBoost.gif (1.1)
- gui/beans/icons/RacedIncrementalLogitBoost\_animated.gif (1.1)
- gui/beans/icons/RandomCommittee.gif (1.1)
- gui/beans/icons/RandomCommittee\_animated.gif (1.1)
- gui/beans/icons/RegressionByDiscretization.gif (1.1)
- gui/beans/icons/RegressionByDiscretization\_animated.gif (1.1)
- gui/beans/icons/Stacking.gif (1.1)
- gui/beans/icons/StackingC.gif (1.1)
- gui/beans/icons/StackingC\_animated.gif (1.1)
- gui/beans/icons/Stacking\_animated.gif (1.1)
- gui/beans/icons/ThresholdSelector.gif (1.1)
- gui/beans/icons/ThresholdSelector\_animated.gif (1.1)
- gui/beans/icons/Vote.gif (1.1)
- gui/beans/icons/Vote\_animated.gif (1.1)

2005-10-28 01:20 mhall

```
Updates from Jeffrey
```

- gui/beans/icons/LeastMedSq.gif (1.2)
- gui/beans/icons/LeastMedSq\_animated.gif (1.2)
- gui/beans/icons/LinearRegression.gif (1.2)
- gui/beans/icons/LinearRegression\_animated.gif (1.2)
- gui/beans/icons/Logistic.gif (1.2)
- gui/beans/icons/Logistic\_animated.gif (1.2)
- gui/beans/icons/PaceRegression.gif (1.2)
- gui/beans/icons/PaceRegression\_animated.gif (1.2)
- gui/beans/icons/RBFNetwork.gif (1.2)
- gui/beans/icons/RBFNetwork\_animated.gif (1.2)
- gui/beans/icons/SMO.gif (1.2)
- gui/beans/icons/SMO\_animated.gif (1.2)
- gui/beans/icons/SMOreg.gif (1.2)
- gui/beans/icons/SMOreg\_animated.gif (1.2)
- gui/beans/icons/SimpleLinearRegression.gif (1.2)
- gui/beans/icons/SimpleLinearRegression\_animated.gif (1.2)

2005-10-25 02:47 fracpete

```
if the classifier throws an exception that contains "not in classpath" (meaning, that some classes are missing) the tests will also be acceptable, e.g., for LibSVM
```

- classifiers/CheckClassifier.html (1.18)

2005-10-25 01:15 fracpete

```
uses now GenericObjectEditor.registerEditors() to register PropertyEditors
```

- gui/explorer/AssociationsPanel.html (1.19)
- gui/explorer/ClassifierPanel.html (1.85)
- gui/explorer/ClustererPanel.html (1.48)
- gui/explorer/DataGeneratorPanel.html (1.2)
- gui/explorer/PreprocessPanel.html (1.56)

2005-10-25 01:14 fracpete

```
uses now GenericObjectEditor.registerEditors() to register PropertyEditors
```

- gui/experiment/AlgorithmListPanel.html (1.14)
- gui/experiment/SetupPanel.html (1.29)

2005-10-25 01:14 fracpete

```
uses now GenericObjectEditor.registerEditors() to register PropertyEditors
```

- gui/beans/AssociatorCustomizer.html (1.3)
- gui/beans/ClassifierCustomizer.html (1.9)
- gui/beans/ClustererCustomizer.html (1.3)
- gui/beans/FilterCustomizer.html (1.7)
- gui/beans/LoaderCustomizer.html (1.11)
- gui/beans/SaverCustomizer.html (1.5)

2005-10-25 01:13 fracpete

```
uses now GenericObjectEditor.registerEditors() to register PropertyEditors
```

- gui/GenericArrayEditor.html (1.15)
- gui/PropertySelectorDialog.html (1.7)
- gui/SelectedTagEditor.html (1.7)

2005-10-25 01:12 fracpete

```
uses now GenericObjectEditor.registerEditors() to register PropertyEditors
```

- classifiers/trees/UserClassifier.html (1.21)

2005-10-21 22:41 fracpete

```
wrapper class for libsvm
based on code from Yasser EL-Manzalawy
uses only reflection to be compileable without the libsvm classes present in the classpath
```

- classifiers/functions/LibSVM.html (1.1)

2005-10-21 22:07 fracpete

```
added little framework for usage from commandline
```

- core/stemmers/IteratedLovinsStemmer.html (1.3)
- core/stemmers/LovinsStemmer.html (1.3)
- core/stemmers/NullStemmer.html (1.3)
- core/stemmers/Stemming.html (1.1)

2005-10-21 04:08 fracpete

```
added Stemmers
```

- gui/GenericObjectEditor.props (1.111)
- gui/GenericPropertiesCreator.props (1.6)

2005-10-21 04:04 fracpete

```
added Serializable
```

- core/stemmers/Stemmer.html (1.2)

2005-10-20 03:21 cvs\_rrb

```
Fixed initialization bug
```

- classifiers/bayes/net/search/global/TAN.html (1.3)

2005-10-20 03:20 cvs\_rrb

```
Fixed initialization bug
```

- classifiers/bayes/net/search/local/TAN.html (1.3)

2005-10-19 20:48 fracpete

```
updated imports for Vector classes
```

- classifiers/functions/PaceRegression.html (1.3)

2005-10-19 20:45 fracpete

```
removed duplicate Matrix/Maths code and moved Vector/Format classes to weka.core.matrix package
```

- classifiers/functions/pace/DoubleVector.html (1.4)
- classifiers/functions/pace/ExponentialFormat.html (1.4)
- classifiers/functions/pace/FlexibleDecimalFormat.html (1.4)
- classifiers/functions/pace/FloatingPointFormat.html (1.4)
- classifiers/functions/pace/IntVector.html (1.3)
- classifiers/functions/pace/Maths.html (1.4)
- classifiers/functions/pace/Matrix.html (1.4)

2005-10-19 20:44 fracpete

```
now use the weka.core.matrix package
```

- classifiers/functions/pace/ChisqMixture.html (1.2)
- classifiers/functions/pace/DiscreteFunction.html (1.2)
- classifiers/functions/pace/MixtureDistribution.html (1.2)
- classifiers/functions/pace/NormalMixture.html (1.2)
- classifiers/functions/pace/PaceMatrix.html (1.2)

2005-10-19 20:43 fracpete

```
added functionality from removed Maths class in weka.classifiers.functions.pace
```

- core/matrix/Maths.html (1.2)

2005-10-19 20:42 fracpete

```
content and col/row made protected for weka.classifiers.functions.pace.PaceMatrix
```

- core/matrix/Matrix.html (1.4)

2005-10-19 20:41 fracpete

```
moved here from weka.classifiers.functions.pace
reason: duplicate Matrix code
```

- core/matrix/DoubleVector.html (1.1)
- core/matrix/ExponentialFormat.html (1.1)
- core/matrix/FlexibleDecimalFormat.html (1.1)
- core/matrix/FloatingPointFormat.html (1.1)
- core/matrix/IntVector.html (1.1)

2005-10-19 02:14 fracpete

```
fixed memory leak
added garbage collector call
```

- gui/ResultHistoryPanel.html (1.21)

2005-10-17 03:20 fracpete

```
fixed handling of inherited options
added markovBlanketClassifier support (made methods public + option -mbc)
```

- classifiers/bayes/net/search/local/LocalScoreSearchAlgorithm.html (1.2)

2005-10-17 03:19 fracpete

```
removed unnecessary if-statements in getOptions
```

- classifiers/bayes/net/search/local/LAGDHillClimber.html (1.4)

2005-10-17 03:18 fracpete

```
removed if-statement for m_nMaxNrOfParents (wrong value anyway)
fixed handling of inherited options
```

- classifiers/bayes/net/search/local/K2.html (1.3)

2005-10-17 03:17 fracpete

```
removed if-statement for m_nMaxNrOfParents (wrong value anyway)
```

- classifiers/bayes/net/search/local/HillClimber.html (1.5)

2005-10-17 03:16 fracpete

```
removed if-statement for m_nMaxNrOfParents (wrong value anyway)
```

- classifiers/bayes/net/search/global/K2.html (1.3)

2005-10-17 03:16 fracpete

```
removed if-statement for m_nMaxNrOfParents (wrong value anyway)
```

- classifiers/bayes/net/search/global/HillClimber.html (1.5)

2005-10-17 03:15 fracpete

```
fixed handling of inherited options
added markovBlanketClassifier support (made methods public + option -mbc)
```

- classifiers/bayes/net/search/global/GlobalScoreSearchAlgorithm.html (1.6)

2005-10-17 03:13 fracpete

```
added get/set/listOptions methods for -cardinality parameter
```

- classifiers/bayes/net/search/ci/ICSSearchAlgorithm.html (1.4)

2005-10-17 03:13 fracpete

```
fixed handling of inherited options
```

- classifiers/bayes/net/search/fixed/FromFile.html (1.5)

2005-10-17 03:12 fracpete

```
changed markovBlanketClassifier methods to protected since derived classes have to implement that behavior
```

- classifiers/bayes/net/search/SearchAlgorithm.html (1.5)

2005-10-17 03:11 fracpete

```
added get/set/listOptions
added initCPTs call in method estimateCPTs (fixes NullPointerEx)
estimateCPTs method now generates correct nominal binary attributes
```

- classifiers/bayes/net/estimate/MultiNomialBMAEstimator.html (1.6)

2005-10-17 03:10 fracpete

```
added get/set/listOptions
added initCPTs call in method estimateCPTs (fixes NullPointerEx)
```

- classifiers/bayes/net/estimate/BMAEstimator.html (1.5)

2005-10-17 03:08 fracpete

```
parser now validates and normalizes
modified retrieval of FOR/TABLE nodes
```

- classifiers/bayes/net/BIFReader.html (1.8)

2005-10-11 19:44 fracpete

```
fixed casting bug in case sum(classProbability)=0 in distributionForInstance
fixed javadoc/revision tag
removed unnecessary imports
```

- classifiers/lazy/KStar.html (1.3)

2005-10-04 20:53 mhall

```
Name fixes
```

- gui/beans/icons/AODE.gif (1.2)
- gui/beans/icons/AODE\_animated.gif (1.2)
- gui/beans/icons/IB1.gif (1.3)
- gui/beans/icons/IB1\_animated.gif (1.3)
- gui/beans/icons/M5P.gif (1.2)
- gui/beans/icons/M5P\_animated.gif (1.2)

2005-09-30 04:49 mhall

```
branches:  1.1.2;
file Id3_animated.gif was initially added on branch book2ndEd-branch.
```

- gui/beans/icons/Id3\_animated.gif (1.1)

2005-09-30 04:49 mhall

```
branches:  1.1.2;
file Id3.gif was initially added on branch book2ndEd-branch.
```

- gui/beans/icons/Id3.gif (1.1)

2005-09-30 04:26 eibe

```
Added tests for empty datasets
```

- classifiers/meta/nestedDichotomies/ClassBalancedND.html (1.2)
- classifiers/meta/nestedDichotomies/DataNearBalancedND.html (1.2)
- classifiers/meta/nestedDichotomies/ND.html (1.2)

2005-09-30 04:23 eibe

```
Added tests for empty datasets
```

- classifiers/meta/END.html (1.2)

2005-09-30 02:14 mhall

```
branches:  1.1.2;
New icons from Jeffrey
```

- gui/beans/icons/ADTree.gif (1.1)
- gui/beans/icons/ADTree\_animated.gif (1.1)
- gui/beans/icons/BayesNet.gif (1.1)
- gui/beans/icons/BayesNet\_animated.gif (1.1)
- gui/beans/icons/ComplementNaiveBayes.gif (1.1)
- gui/beans/icons/ComplementNaiveBayes\_animated.gif (1.1)
- gui/beans/icons/DecisionStump.gif (1.1)
- gui/beans/icons/DecisionStump\_animated.gif (1.1)
- gui/beans/icons/ID3.gif (1.1)
- gui/beans/icons/ID3\_animated.gif (1.1)
- gui/beans/icons/KStar.gif (1.1)
- gui/beans/icons/KStar\_animated.gif (1.1)
- gui/beans/icons/LBR.gif (1.1)
- gui/beans/icons/LBR\_animated.gif (1.1)
- gui/beans/icons/LMT.gif (1.1)
- gui/beans/icons/LMT\_animated.gif (1.1)
- gui/beans/icons/LWL.gif (1.1)
- gui/beans/icons/LWL\_animated.gif (1.1)
- gui/beans/icons/MultilayerPerceptron.gif (1.1)
- gui/beans/icons/MultilayerPerceptron\_animated.gif (1.1)
- gui/beans/icons/NBTree.gif (1.1)
- gui/beans/icons/NBTree\_animated.gif (1.1)
- gui/beans/icons/NaiveBayes.gif (1.1)
- gui/beans/icons/NaiveBayesMultinomial.gif (1.1)
- gui/beans/icons/NaiveBayesMultinomial\_animated.gif (1.1)
- gui/beans/icons/NaiveBayesSimple.gif (1.1)
- gui/beans/icons/NaiveBayesSimple\_animated.gif (1.1)
- gui/beans/icons/NaiveBayesUpdateable.gif (1.1)
- gui/beans/icons/NaiveBayesUpdateable\_animated.gif (1.1)
- gui/beans/icons/NaiveBayes\_animated.gif (1.1)
- gui/beans/icons/REPTree.gif (1.1)
- gui/beans/icons/REPTree\_animated.gif (1.1)
- gui/beans/icons/RandomForest.gif (1.1)
- gui/beans/icons/RandomForest\_animated.gif (1.1)
- gui/beans/icons/RandomTree.gif (1.1)
- gui/beans/icons/RandomTree\_animated.gif (1.1)
- gui/beans/icons/SimpleLogistic.gif (1.1)
- gui/beans/icons/SimpleLogistic\_animated.gif (1.1)
- gui/beans/icons/UserClassifier.gif (1.1)
- gui/beans/icons/UserClassifier\_animated.gif (1.1)
- gui/beans/icons/VotedPerceptron.gif (1.1)
- gui/beans/icons/VotedPerceptron\_animated.gif (1.1)
- gui/beans/icons/Winnow.gif (1.1)
- gui/beans/icons/Winnow\_animated.gif (1.1)

2005-09-30 02:11 mhall

```
Updated icons thanks to Jeffrey
```

- gui/beans/icons/IBk.gif (1.2)
- gui/beans/icons/IBk\_animated.gif (1.2)
- gui/beans/icons/J48.gif (1.2)
- gui/beans/icons/J48\_animated.gif (1.2)
